# Supplementary material for: What does good care look like to people living with congenital heart disease in the 21st century? Qualitative online, asynchronous discussion forums
Source: BMJ Open. 2024 Jul 1;14(7):e079691. doi: 10.1136/bmjopen-2023-079691 (PMC11218017; doi:10.1136/bmjopen-2023-079691)
Supplement: Author's manuscript [file bmjopen-2023-079691.draft_revisions.pdf]

BMJ Open

BMJ Open is committed to open peer review. As part of this commitment we make the peer review history of every article we publish publicly available.

When an article is published we post the peer reviewers' comments and the authors' responses online. We also post the versions of the paper that were used during peer review. These are the versions that the peer review comments apply to.

The versions of the paper that follow are the versions that were submitted during the peer review process. They are not the versions of record or the final published versions. They should not be cited or distributed as the published version of this manuscript.

BMJ Open is an open access journal and the full, final, typeset and author-corrected version of record of the manuscript is available on our site with no access controls, subscription charges or pay-per-view fees (http://bmjopen.bmj.com).

If you have any questions on BMJ Open's open peer review process please email info.bmjopen@bmj.com

Living with congenital heart disease in the 21st century: what does good care look like to patients?

Journal:	*BMJ Open*
Manuscript ID	bmjopen-2023-079691
Article Type:	Original research
Date Submitted by the Author:	08-Sep-2023
Complete List of Authors:	Wray, Jo; Great Ormond Street Hospital for Children NHS Foundation Trust, Heart and Lung Directorate; University College London<br>Pagel, Christina; University College London, Clinical Operational Research Unit, Department of Mathematics<br>Coats, Louise; Freeman Hospital, ; Newcastle University,<br>Chester, Adrian; Heart Science Centre<br>Kennedy, Fiona; Barts Health NHS Trust<br>Crowe, Sonya; University College London, Clinical Operational Research Unit, Department of Mathematics
Keywords:	Quality in health care < HEALTH SERVICES ADMINISTRATION & MANAGEMENT, Congenital heart disease < CARDIOLOGY, Social Media

SCHOLARONE™
Manuscripts

I, the Submitting Author has the right to grant and does grant on behalf of all authors of the Work (as defined in the below author licence), an exclusive licence and/or a non-exclusive licence for contributions from authors who are: i) UK Crown employees; ii) where BMJ has agreed a CC-BY licence shall apply, and/or iii) in accordance with the terms applicable for US Federal Government officers or employees acting as part of their official duties; on a worldwide, perpetual, irrevocable, royalty-free basis to BMJ Publishing Group Ltd ("BMJ") its licensees and where the relevant Journal is co-owned by BMJ to the co-owners of the Journal, to publish the Work in this journal and any other BMJ products and to exploit all rights, as set out in our licence.

The Submitting Author accepts and understands that any supply made under these terms is made by BMJ to the Submitting Author unless you are acting as an employee on behalf of your employer or a postgraduate student of an affiliated institution which is paying any applicable article publishing charge ("APC") for Open Access articles. Where the Submitting Author wishes to make the Work available on an Open Access basis (and intends to pay the relevant APC), the terms of reuse of such Open Access shall be governed by a Creative Commons licence – details of these licences and which Creative Commons licence will apply to this Work are set out in our licence referred to above.

Other than as permitted in any relevant BMJ Author's Self Archiving Policies, I confirm this Work has not been accepted for publication elsewhere, is not being considered for publication elsewhere and does not duplicate material already published. I confirm all authors consent to publication of this Work and authorise the granting of this licence.

For peer review only

Living with congenital heart disease in the 21st century: what does good care look like to patients?

Jo Wray[1,2], Christina Pagel[3], Louise Coats[4], Adrian H. Chester[5], Fiona Kennedy[6] and Sonya Crowe[3]

[1] Heart and Lung Directorate, Great Ormond Street Hospital for Children NHS Foundation Trust, London, UK

[2] Institute of Cardiovascular Science, University College London, UK

[3] Clinical Operational Research Unit, University College London, UK

[4] The Newcastle upon Tyne Hospitals NHS Foundation Trust, Newcastle, UK

[5]The Magdi Yacoub Institute, Heart Science Centre, Harefield, UK

[6] Barts Health NHS Trust, London, UK

Address for correspondence:
Dr Jo Wray
Great Ormond Street Hospital for Children NHS Foundation Trust
London
WC1N 3JH
Email: jo.wray@gosh.nhs.uk

For peer review only

Abstract

Objectives

As part of a wider study, our aim was to elicit perspectives of patients with congenital heart disease (CHD) and/or their parents/carers about their experiences of health care and what is important to them when receiving care.

Methods

Three patient charities (Little Hearts Matter, the Somerville Foundation and the Children's Heart Federation) each set up and moderated one or more closed, asynchronous, online discussion groups via their Facebook pages. Separate forums were facilitated for adult patients (and carers), adolescent patients and parents/carers of children and young people with CHD. Questions focused on experiences of inpatient and outpatient care and what 'good' care looked like. Forums ran for 12-24 weeks and a single transcript was generated from each forum. All transcripts were anonymised and thematically analysed.

Results

Five forums were run across the three charities, and 343 participants signed up to the forums. Six linked themes related to processes of care were identified: relationships, communication, access, coordination, episodes of care and holistic care. These impacted how care was experienced and, indirectly, outcomes of CHD and its treatment. In addition, context relating to stages of the patient journey were described, together with patient-related factors such as patients' knowledge and expertise in their own condition.

Conclusions

Patients with CHD and their parents/carers want individualised, person-centred care delivered within an appropriately resourced, multidisciplinary service. Although examples of excellent care

For peer review only

were provided it is evident that, from the perspective of patients and parents/carers, some NHS Standards for patients with CHD were not being met.

Strengths and limitations

- Data collection methods (asynchronous discussion forums on Facebook) facilitated accessibility for participants and resulted in a large sample of geographically and clinically diverse participants of different ages
- Use of social media to collect data supports participation by those who might otherwise not engage in research
- Most participants were female and ethnic diversity was limited
- The study design precluded participation from those who could not read English, use social media or were unfamiliar with, or without access to, appropriate technology

For peer review only

Background

Successive improvements in surgical and medical care mean many more children with congenital heart disease (CHD) now reach adulthood and live longer adult lives.(1, 2) Furthermore, increasing numbers live with significant physical and mental health co-morbidities alongside the natural progression of their CHD.(3-6) The vast majority of this heterogeneous group are advised to remain under specialist life-long follow-up (7, 8) though sporadic clinic non-attendance is frequent, particularly in adulthood (9), and patients may be lost to follow-up entirely, sometimes with significant consequences (10). Unlike many other chronic conditions, requirement for specialist follow-up means patients with CHD usually remain under the care of the team at the tertiary hospital, which may be far from where they live. Many will require investigations that may include unanticipated invasive procedures. (9) Some may be seen in outreach clinics, usually by members of the specialist team but also by non-CHD cardiovascular specialists.(11) Occasionally, patients with CHD remain undiagnosed until adulthood(12, 13) when they typically present with symptoms.

The way in which patients (and their carers) experience care has a significant impact on their engagement with that care and the professionals delivering it.(14) In the National Health Service (NHS) Outcomes Framework(15) 'ensuring that people have a positive experience of care' was identified as a priority, recognising the consequences for safety and effectiveness. However, if the NHS is to provide optimal care that meets the needs and expectations of service users, it is important to understand what characterises 'good' care from the patient and their family's perspective.

In this study, we elicited the perspectives of patients and carers about their experiences of CHD care. This was carried out as part of a wider study (Congenital Heart Audit: Measuring Progress In Outcomes Nationally (CHAMPION))(16) aiming to develop tools for routine measurement of outcomes in CHD that are considered relevant to all stakeholders.

For peer review only

Methods

Design

A qualitative approach underpinned by an interpretivist framework was used, in which participant (patient or parent/carer) views were elicited through online discussion forums.

Patient and Public Involvement (PPI)

A patient co-researcher (AC) led a PPI group for the overall study (comprising three adults with CHD and one grandparent of a child with CHD). The PPI group reviewed and provided feedback on the content and language of the questions for the online forum and on the findings prior to submission. The forum questions and the presentation of the findings were revised based on feedback from the PPI group.

Participants and data collection

We adopted an established approach. (17, 18) (19) Three UK CHD charities (Little Hearts Matter; Children's Heart Federation; Somerville Foundation) facilitated and moderated one or more closed, asynchronous, online discussion groups via their Facebook pages. This enabled us to elicit a range of views (parents/carers of younger children, adolescent and adult patients) across the spectrum of CHD severity (complex/less complex). The research team developed questions and revised their content and language based on feedback from the PPI group and charity collaborators. On the advice of the charities, separate forums were run for adult patients, adolescent patients and parents/carers of young people with CHD. Carers of adult patients could participate in the adult discussion groups. The discussion forums were advertised by each charity on their web page with potential participants signposted to the charity's Facebook page for further information about the study. People wanting to participate were asked to provide basic demographic details and

For peer review only

subsequently directed to the appropriate closed Facebook group where they could join the discussion. The charities could introduce prompts or post new questions, based on responses. Questions for each forum were similar, with minor wording revisions to reflect the respondent group (Table 1). The forums ran for 12-24 weeks (dependent on responses and interruptions due to Covid-19).

Data management and analysis

Identifiable data were removed by the charities before transfer of the anonymised transcript with aggregated demographics to the research team. Transcripts were thematically analysed(20) independently by members of the research team: codes were given to segments of data and similar codes grouped to create themes and subthemes related to experience of CHD care. The researchers met to discuss and finalise the themes. The themes and transcripts were sent to an additional member of the research team (not involved in the coding) to review (FK) to ensure that all data about experience of care were accurately represented in the themes.

Ethics

The study was granted ethical approval by the North of Scotland Research Ethics Service (ref: 20/NS/0022).

Results

Five forums were run across the three charities. Demographics of participants are shown in Table 2.

Six linked themes related to processes of care were identified: relationships, communication, access, coordination, episodes of care and holistic care. These impacted how care was experienced and, indirectly, outcomes of CHD and its treatment. In addition, context relating to stages of the patient

For peer review only

journey and patient-related factors eg. patients' expertise in their own condition were described (Figure 1). Themes are described below, with illustrative quotes.

Relationships

Participants discussed the importance of the patient-clinician relationship in terms of the characteristics of the clinician and of the relationship. Patients want to have a '*great relationship with [the] consultant, knowing you'll see someone who 'knows' you and your condition really well',* and emphasised the need for awareness of, and continuity in, those providing care: *"They don't have to be my mate but it's important that they're cognisant of the context because CHD is lifelong and affects all parts of my life."* The importance of professionals being kind, approachable, honest and caring as well as being excellent communicators and expert in CHD were highlighted. Participants felt they needed a team they respect and trust, who listen and treat them "*like a human being and not a burden on resources. The impact of being told that - both overtly and implicitly - is severe, and affects engagement"*' A parent of a child said of the surgeon: *"His calm, kind, confident communication and how he took personal responsibility was pivotal in giving me the confidence to trust him to operate on my son."* Another parent said*: "The best care we have received has been when we were listened to and not judged for the questions and requirements we had."* Other patients described the importance of being able to *"maintain a great relationship with the CNS* [clinical nurse specialist] *nurses as often it is hard to remember or say everything needed to the consultant,"* with the need for others to advocate for them when they feel unable to do so also acknowledged. Recognition that adult patients are experts in their own care and need to feel empowered and believed was highlighted, with emphasis on the importance of feeling part of the team: *"I like the feeling that my medical care is a partnership between the cardiac team (who know about hearts) and me (I know about me and what's normal/ok/priority for me)."* Another patient commented: *"I hope the team understands a little of what I've been through, what I bring with me in terms of experience, knowledge but also emotionally."* Participants also discussed the importance of

For peer review only

being involved in decision making, *"What's important to me is that all my team totally understand my condition, listen to me and include me in any decisions".* One participant talked about being able to make *"Self-referrals, avoiding local cardiac care, we know when we feel shitty".*

Communication

Practical aspects of communication both with and between professionals were discussed in terms of functional elements (accuracy, timeliness), content (e.g. information about procedures and associated risks) and the mechanisms of communication: *"…results from tests being given by consultant not just a short letter that is sent to GP".* They also talked about the importance of being given honest feedback: *"I'd rather have the detail with an explanation about what this means even though it might be difficult to come to terms with. I want to be making informed decisions."*

Many patients with CHD are under the care of multiple teams and specialists. Participants described the importance (and often failure) of communication to ensure joined up care – *"Could that all have been avoided if the surgical team had involved cardiology more? Better communication certainly would have helped either way"* – and discussed communication between teams – "*why do the ACHD team and the EP team not seem to talk to one another? What's the point of coming up with a plan with the one team and then the other walking in and saying something different?".* They also described challenges in communication between the specialist centres and outreach and primary care services. As one participant said, *"I'm seen at an outreach clinic and there is quite often a lack of communication between the outreach clinic and the main hospital"* whilst another described the importance of there being "*a note on the GP system so that when you appear with heart symptoms they take it seriously".*

Parents of paediatric patients discussed the importance of communication with schools and nurseries and getting help with education, health and care plans: *"Schools always panic over*

For peer review only

everything. My son's first week back at school I was called 3 times by the matron due to little things. I asked cardiac nurses for info for school and was just given general information leaflets. They should be more person centred as 2 kids with the same condition will be at different stages and have different symptoms."

Access and coordination

Respondents described challenges accessing care when they were moving to another hospital, out of hours or when they were unwell or needed advice: *"Knowing that I can ask a question or get a query answered quickly is very important to me. So, timely and accessible care is important*". Some patients had been lost to follow-up: "*my checkups were not continued…notes must have been lost somewhere. I had no drugs, nothing and I had a stroke which I believe could have been avoided"* or had to fight to get treatment: *"Actually getting any care at all would be good… I haven't been referred to the ACHD team despite asking and my 'care' has been non-existent".* Others talked about "*being lost in the system…never followed-up*". In contrast, some parents talked about the ready access they had to the clinical team or an open-door policy to access the ward if their child was unwell: *"My son is still looked after [by] a multidisciplinary team over two hospitals and we have 24 hour access to a local hospital that also have wonderful and caring doctors. My GP called us at the various hospitals to check we were ok. I feel very privileged to have such a joined-up way of all the hospitals and primary care teams working together for my son's health."*

Coordination of care was mentioned frequently, particularly in relation to appointments and investigations and organising them to happen at the same time ("*Appointments in a cluster – MRI, echo etc so we don't have multiple trips to manage"),* with some highlighting examples of good practice: *"In recent years (pre Covid) it has gone like clockwork and I'm usually in hospital for a couple of hours at most."* Others talked about the complexity of their problems and needing everything to be together: "*I would like somewhere to go where all our older problems are taken*

For peer review only

care of… heart, everything under one roof not non-guch [ACHD] hosp where docs don't understand your condition". Another respondent said: "*Definitely not having consultant appointment- test arranged for 2 months later, another test a couple of months after that and because of that result another test after that with a consultation several months after that which takes you a year down the line to then have a consultation with someone who hasn't even looked at the results of the tests!"*

Episodes of care

Participants discussed their experiences of clinics and inpatient stays, describing the importance of being given enough time (*"appointments not to be rushed"),* their experiences of undergoing tests and the people conducting them, and what makes attending difficult. Changes to clinic arrangements made as a result of Covid-19 were viewed positively by some: "*What has been useful in Covid times is going for tests and then having a video or telephone conversation to discuss those test results. I think this is a good model to take forward*." They also talked about inpatient stays, in terms of the environment, ability to sleep on the ward, available activities (children) and experience of discharge: *"Having a good discharge! Why oh why can it take hours and hours to get out of hospital when you are clinically well enough to leave. Getting out of hospital is as important as being admitted in the first place."* Respondents highlighted the value of being routinely asked about their experiences of outpatient and inpatient care through completion of patient reported experience measures. Linked to this, some adult patients articulated the importance of patients being involved in service development initiatives.

Holistic care

Mental health and the need for psychological support were mentioned by patients and carers as important but often lacking. Some described waiting for psychology support for many years or services not being available at all: *"Access to psychology at clinics - that is non-existent".* Some adult

For peer review only

patients received a late diagnosis, with repercussions for their mental health: *"I found out later in life and I found the mental health support was limited, specifically with adjusting to the news that my heart could have ripped itself apart. Those who are diagnosed later or suddenly escalate should be referred to mental health specialists as a priority."* Another participant who had experienced psychological issues for which they had not received any support commented: *"My story exposed the importance of psychological care and the potential physical damage that can be caused to the cardiological system from stress, anxiety and childhood trauma instigated by CHD."* The importance of other professionals in the wider team was also identified, including for children – *"I think that every child that had life-long illness should have regular play therapy, to help deal with their fear and emotions they face."* Finally, the valuable role of patient support organisations was acknowledged, *"They put me in touch with [charity], who have been my saviour."*

Discussion

This study, which reports CHD patients' and their carers' perspectives on their experiences of care, identifies key themes (relationships, communication, access and coordination, episodes of care and holistic care) which are important to patient experience and outcomes. It suggests a number of the NHS CHD recommended standards (23) are presently not being met, e.g. psychology provision and referral to ACHD services – although some (particularly parents) gave examples of excellent care.

Building on the conceptual map of Entwistle (21) who undertook a critical interpretive synthesis of research literature on patients' perspectives of health care delivery, we have used our findings to characterise what 'good care' looks like for CHD patients and their carers (Figure 2). This approach recognises that the characteristics of health services and actions of professionals impact how patients (and families) experience and engage with care and our findings resonate with previous studies.(22) CHD patients are a unique group due to their complexity, the requirement for life-long care and the diversity in how and by whom care is delivered. The NHS Standards for patients with

For peer review only

CHD(23) clearly outline what centres should provide in the way of services and access to professionals and this study builds on these by offering insight from the service users' perspective.

Limitations

Data collection (asynchronous discussion forums on Facebook) facilitated accessibility for participants and resulted in geographically and clinically diverse participants of different ages. However, a broad range of ethnicities was not seen and the vast majority of participants were female, supporting previous findings that females are more likely to use social media.(24, 25) The study design precluded participation from those who could not read English, use social media or were unfamiliar with, or without access to, appropriate technology and potentially those with intellectual disability. Patients and families already in contact with the charities may have been more likely to engage leading to potential selection bias and limited generalisability of the findings. Due to the format of the data provided we were unable to link demographic characteristics to responses. Finally, some adult patients experienced elements of the patient journey some time ago and their experiences may not be reflective of how some of these aspects of care are managed today.

The fourth principle of the NHS Constitution of England(26) states that 'the patient will be at the heart of everything the NHS does' and that 'NHS services must reflect, and should be coordinated around and tailored to, the needs and preferences of patients, their families and their carers.' Delivering good care and a good patient experience are crucial if patients are to engage, and remain engaged, with their health care and outcomes are to be optimised;(27) understanding what 'good' care means to patients and families is a key element of that process. Our findings, whilst indicating that patients with CHD share commonalities with other patient groups, also reflect the challenges of delivering comprehensive care to this diverse and growing population. Participants have articulated how they would like care to be delivered and what good care looks like, with an emphasis on

For peer review only

individualised, person-centred care delivered within an appropriately resourced, multidisciplinary service. They want to be acknowledged as experts in their own condition and for the whole life impact of CHD and its treatment to be recognised, not just the physical impact. It is now important that these findings are used to inform quality improvement initiatives or, where this remains aspirational, to manage patients' and families' expectations. The results could also be used to inform the development of patient reported experience measures for the CHD population, the routine completion of which would enable services to be continually evaluated, benchmarked and improved, with benefits for optimising patients' experiences and outcomes.

Acknowledgements

The authors thank The Somerville Foundation, Little Hearts Matter and the Children's Heart Federation for recruiting participants and moderating the forums, and for their contributions, together with the PPI group, to the development and review of the forum questions.

Research at Great Ormond Street Hospital is supported by the Great Ormond Street Hospital NIHR Biomedical Research Centre.

Funding statement

This study is independent research funded by the National Institute for Health Research (Policy Research Programme, Congenital Heart Audit: Measuring Progress In Outcomes Nationally (CHAMPION), PR-R20-0318-23001). The views expressed in this publication are those of the author(s) and not necessarily those of the NHS, the National Institute for Health Research or the Department of Health and Social Care.

For peer review only

References

1. Global Burden of Disease Congenital Heart Disease Collaborators. Global, regional, and national burden of congenital heart disease, 1990-2017: a systematic analysis for the Global Burden of Disease Study 2017. Lancet Child Adolesc Health. 2020;4(3):185-200.

2. Benziger CP, Stout K, Zaragoza-Macias E, Bertozzi-Villa A, Flaxman AD. Projected growth of the adult congenital heart disease population in the United States to 2050: an integrative systems modeling approach. Popul Health Metr. 2015;13:29.

3. Desai R, Patel K, Dave H, Shah K, DeWitt N, Fong HK, et al. Nationwide Frequency, Sequential Trends, and Impact of Co-morbid Mental Health Disorders on Hospitalizations, Outcomes, and Healthcare Resource Utilization in Adult Congenital Heart Disease. Am J Cardiol. 2020;125(8):1256-62.

4. El-Chouli M, Meddis A, Christensen DM, Gerds TA, Sehested T, Malmborg M, et al. Lifetime risk of comorbidity in patients with simple congenital heart disease: a Danish nationwide study. Eur Heart J. 2022.

5. Brida M, Lovric D, Griselli M, Riesgo Gil F, Gatzoulis MA. Heart failure in adults with congenital heart disease. Int J Cardiol. 2022;357:39-45.

6. Alonso-Gonzalez R, Borgia F, Diller GP, Inuzuka R, Kempny A, Martinez-Naharro A, et al. Abnormal lung function in adults with congenital heart disease: prevalence, relation to cardiac anatomy, and association with survival. Circulation. 2013;127(8):882-90.

7. Kempny A, Diller GP, Dimopoulos K, Alonso-Gonzalez R, Uebing A, Li W, et al. Determinants of outpatient clinic attendance amongst adults with congenital heart disease and outcome. Int J Cardiol. 2016;203:245-50.

For peer review only

8. Baumgartner H, Budts W, Chessa M, Deanfield J, Eicken A, Holm J, et al. Recommendations for organization of care for adults with congenital heart disease and for training in the subspecialty of 'Grown-up Congenital Heart Disease' in Europe: a position paper of the Working Group on Grown-up Congenital Heart Disease of the European Society of Cardiology. Eur Heart J. 2014;35(11):686-90.

9. Coats L, Chaudhry B. Ambulatory Care in Adult Congenital Heart Disease-Time for Change? J Clin Med. 2022;11(7).

10. Wray J, Frigiola A, Bull C, Adult Congenital Heart disease Research N. Loss to specialist follow-up in congenital heart disease; out of sight, out of mind. Heart. 2013;99(7):485-90.

11. Seidel L, Nebel K, Achenbach S, Bauer U, Ewert P, Freilinger S, et al. Facts about the General Medical Care of Adults with Congenital Heart Defects: Experience of a Tertiary Care Center. J Clin Med. 2020;9(6).

12. De Angelis F, Savino K, Colombo A, Sardone M, Ambrosio G. Never too Grown-Up for a Congenital Heart Disease: Diagnosis of Transitional Atrioventricular Canal in a 50-Year-Old Male. J Cardiovasc Echogr. 2019;29(1):35-8.

13. Sadeghpour A, Kim H, Chamis AL. Undiagnosed Atrial Septal Defect in the Setting of Comorbidities and Ventricular Failure: Seemingly Simple Disease with a Challenging Diagnosis. CASE (Phila). 2023;7(2):72-80.

14. de Silva D. Measuring patient experience. The Health Foundation 2013.

15. Department of Health. NHS Outcomes Framework 2012/13.; 2011.

16. Crowe S, Chester A, Ambler G, Wray J, Brown K, von Klemperer K, et al. Congenital Heart Audit: Measuring Progress In Outcomes Nationally (CHAMPION) 2019 [Available from: https://fundingawards.nihr.ac.uk/award/PR-R20-0318-23001.

For peer review only

17. Rajagopal V, Brown K, Pagel C, Wray J. Parental understanding of our communication of morbidity associated with paediatric cardiac surgery: a qualitative study. BMJ Paediatr Open. 2020;4(1):e000578.

18. Wray J, Brown K, Tregay J, Crowe S, Knowles R, Bull K, et al. Parents' Experiences of Caring for Their Child at the Time of Discharge After Cardiac Surgery and During the Postdischarge Period: Qualitative Study Using an Online Forum. J Med Internet Res. 2018;20(5):e155.

19. Wray J, Pagel C, Chester AH, Kennedy F, Crowe S. What was the impact of the first wave of COVID-19 on the delivery of care to children and adults with congenital heart disease? A qualitative study using online forums. BMJ Open. 2021;11(9):e049006.

20. Braun V, Clarke V. Using thematic analysis in psychology. Qualitative research in psychology. 2006;3:77-101.

21. Entwistle V, Firnigl D, Ryan M, Francis J, Kinghorn P. Which experiences of health care delivery matter to service users and why? A critical interpretive synthesis and conceptual map. J Health Serv Res Policy. 2012;17(2):70-8.

22. Robert G, Cornwell J. Rethinking policy approaches to measuring and improving patient experience. Journal of Health Services Research & Policy. 2013;18(2):67-9.

23. NHS England. Congenital Heart Disease Standards & Specifications 2016 [Available from: https://www.england.nhs.uk/wp-content/uploads/2018/08/Congenital-heart-disease-standards-and-specifications.pdf.

24. Ye L, Chen Y, Cai Y, Kao YW, Wang Y, Chen M, et al. Gender Differences in the Nonspecific and Health-Specific Use of Social Media Before and During the COVID-19 Pandemic: Trend Analysis Using HINTS 2017-2020 Data. J Health Commun. 2023:1-10.

For peer review only

25. Johnson LA, Dias N, Clarkson G, Schreier AM. Social media as a recruitment method to reach a diverse sample of bereaved parents. Appl Nurs Res. 2019;50:151201.

26. Department of Health and Social Care. Guidance - The NHS Constitution for England 2021 [Available from: https://www.gov.uk/government/publications/the-nhs-constitution-for-england/the-nhs-constitution-for-england.

27. Doyle C, Lennox L, Bell D. A systematic review of evidence on the links between patient experience and clinical safety and effectiveness. BMJ Open. 2013;3(1).

For peer review only

Table 1: Examples of questions related to experiences of care for the adult patient forums

Questions for the parent/carer and teenager forums were similar, with minor wording changes to reflect those respondent groups (e.g. designed to appeal to teenagers or wording appropriate for carers rather than patients).

1. Understanding how patients and families judge how 'good' a service is will help us to identify things that services do well and those aspects where the NHS could improve.

    - What do you think makes a service good quality? What sort of things do you want to know about a hospital or service to judge whether it is a good place to care for you?
    - What things might make you worry that the hospital / service was not as good quality as other hospitals / services?

2. We would like to know about what information you think should be collected about people's *experience* of the care they receive – for example, about how health professionals talk to patients and their families.

    - Thinking about some recent care you have had, are there any particular aspects of your experience that the NHS could collect about those services to judge their quality?

3. We would like to ask you about your experience of attending clinic appointments, which we recognise is not always easy for patients. How easy or difficult have you found it to keep up with follow-up appointments? Are there particular things that make it easy or difficult for you to attend follow-up appointments? How easy do you find it to see professionals other than doctors and nurses when you attend clinic, if you want to (such as social workers, psychologists, play specialists)?

For peer review only - http://bmjopen.bmj.com/site/about/guidelines.xhtml

Table 2: Characteristics of those who signed up to the online forums

	Number (%)
Participants: Adults with CHD	235 (69)
Young people with CHD	11 (3)
Parents/carers of adult patients with CHD	7 (2)
Parents/carers of children with CHD	90 (26)
Participant gender: Male	33 (10)
Female	245 (71)
Unknown	65 (19)
Participant age group: <16 years	2 (1)
16-20	11 (3)
21-30	32 (9)
31-40	69 (20)
41-50	72 (21)
51-60	65 (19)
>61 years	15 (4)
Unknown	77 (22)
Age group of person with CHD: 0-1 years	9 (3)
2-5 years	17 (4)
6-10 years	15 (4)
11-15 years	8 (2)
16-18 years	9 (3)
>18 years	238 (69)
Unknown	47 (14)
Participant ethnicity: White	326 (95)
Non-white	5 (1)
Unknown	12 (3)
Location of specialist service: England (North East)	11 (3)
England (North West)	26 (6)
England (Yorkshire and the Humber)	20 (6)
England (East Midlands)	14 (4)
England (West Midlands)	47 (14)
England (East of England)	9 (3)
England (London)	75 (22)
England (South East)	28 (8)
England (South West)	27 (8)
Wales	8 (2)
Scotland	24 (7)
Northern Ireland/other	15 (4)
Unknown/none	39 (11)
Location of home: England (North East)	13 (4)
England (North West)	43 (13)
England (Yorkshire and the Humber)	22 (6)
England (East Midlands)	20 (6)
England (West Midlands)	36 (10)
England (East of England)	23 (7)
England (London)	22 (6)
England (South East)	57 (17)
England (South West)	46 (13)
Wales	21 (6)

For peer review only

Scotland	25 (7)
Northern Ireland/other	8 (2)
Unknown	7 (2)
Complexity of CHD: Single ventricle condition	121 (35)
Biventricular condition	216 (63)
Unknown	6 (2)

A number of participants chose not to provide some or any demographic information

For peer review only

For peer review only

Figure 1: Map of the themes developed from the data

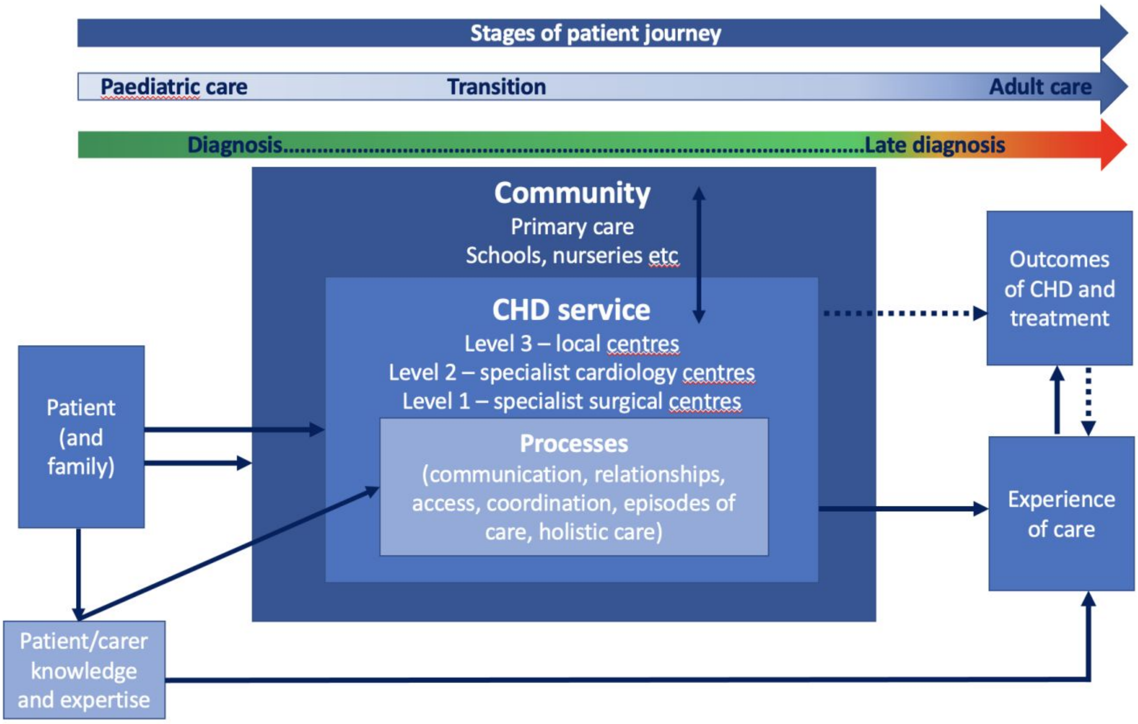

Footnote – dotted lines indicate hypothesised relationships not directly ascertainable from the online forum data

For peer review only

For peer review only

Figure 2: Identified characteristics and actions of health care services and staff and resulting potential for delivery of ‘good’ care

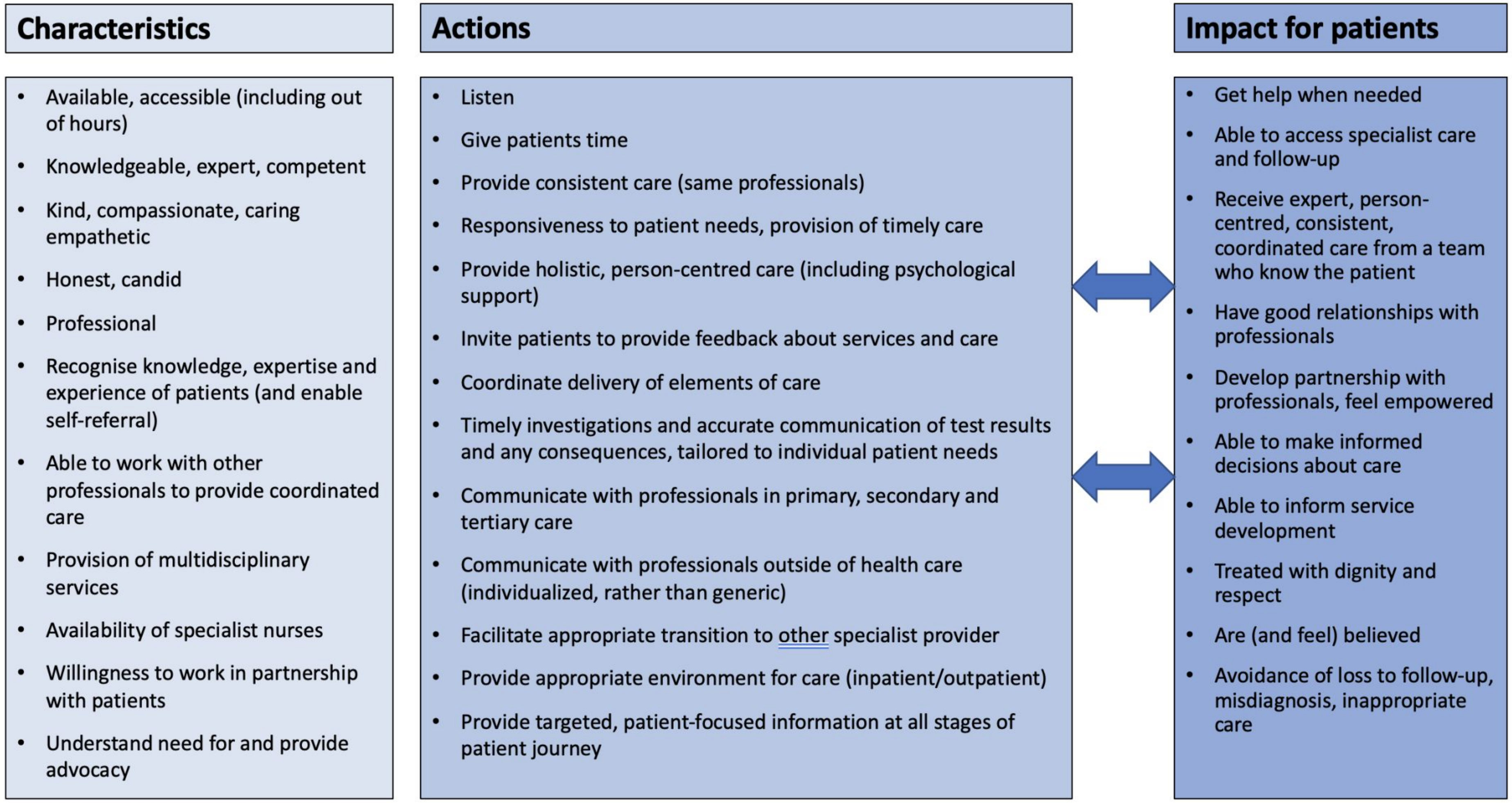

What does good care look like to people living with congenital heart disease in the 21st century? Qualitative online, asynchronous discussion forums

Journal:	*BMJ Open*
Manuscript ID	bmjopen-2023-079691.R1
Article Type:	Original research
Date Submitted by the Author:	26-Apr-2024
Complete List of Authors:	Wray, Jo; Great Ormond Street Hospital for Children NHS Foundation Trust, Heart and Lung Directorate; University College London<br>Pagel, Christina; University College London, Clinical Operational Research Unit, Department of Mathematics<br>Coats, Louise; Freeman Hospital, ; Newcastle University,<br>Chester, Adrian; Heart Science Centre<br>Kennedy, Fiona; Barts Health NHS Trust<br>Crowe, Sonya; University College London, Clinical Operational Research Unit, Department of Mathematics
**Primary Subject Heading**:	Health services research
Secondary Subject Heading:	Communication, Qualitative research
Keywords:	Quality in health care < HEALTH SERVICES ADMINISTRATION & MANAGEMENT, Congenital heart disease < CARDIOLOGY, Social Media

SCHOLARONE™
Manuscripts

I, the Submitting Author has the right to grant and does grant on behalf of all authors of the Work (as defined in the below author licence), an exclusive licence and/or a non-exclusive licence for contributions from authors who are: i) UK Crown employees; ii) where BMJ has agreed a CC-BY licence shall apply, and/or iii) in accordance with the terms applicable for US Federal Government officers or employees acting as part of their official duties; on a worldwide, perpetual, irrevocable, royalty-free basis to BMJ Publishing Group Ltd ("BMJ") its licensees and where the relevant Journal is co-owned by BMJ to the co-owners of the Journal, to publish the Work in this journal and any other BMJ products and to exploit all rights, as set out in our licence.

The Submitting Author accepts and understands that any supply made under these terms is made by BMJ to the Submitting Author unless you are acting as an employee on behalf of your employer or a postgraduate student of an affiliated institution which is paying any applicable article publishing charge ("APC") for Open Access articles. Where the Submitting Author wishes to make the Work available on an Open Access basis (and intends to pay the relevant APC), the terms of reuse of such Open Access shall be governed by a Creative Commons licence – details of these licences and which Creative Commons licence will apply to this Work are set out in our licence referred to above.

Other than as permitted in any relevant BMJ Author's Self Archiving Policies, I confirm this Work has not been accepted for publication elsewhere, is not being considered for publication elsewhere and does not duplicate material already published. I confirm all authors consent to publication of this Work and authorise the granting of this licence.

For peer review only

What does good care look like to people living with congenital heart disease in the 21st century? Qualitative online, asynchronous discussion forums

Jo Wray[1,2], Christina Pagel[3], Louise Coats[4], Adrian H. Chester[5], Fiona Kennedy[6] and Sonya Crowe[3]

[1] Heart and Lung Directorate, Great Ormond Street Hospital for Children NHS Foundation Trust, London, UK

[2] Institute of Cardiovascular Science, University College London, UK

[3] Clinical Operational Research Unit, University College London, UK

[4] The Newcastle upon Tyne Hospitals NHS Foundation Trust, Newcastle, UK

[5] The Magdi Yacoub Institute, Heart Science Centre, Harefield, UK

[6] Barts Health NHS Trust, London, UK

Address for correspondence:
Dr Jo Wray
Great Ormond Street Hospital for Children NHS Foundation Trust
London
WC1N 3JH
Email: jo.wray@gosh.nhs.uk

For peer review only

Abstract

Objectives

As part of a wider study, our aim was to elicit perspectives of people with congenital heart disease (CHD) and/or their parents/carers about their experiences of health care and what is important to them when receiving care.

Design and setting

A qualitative study involving a series of closed, asynchronous, online discussion forums underpinned by an interpretivist framework and set up and moderated by three patient charities via their Facebook pages.

Participants

People with congenital heart disease and parents/carers of people with congenital heart disease from the United Kingdom.

Results

Five forums were run for 12-24 weeks across the three charities, and 343 participants signed up to the forums. Four linked themes related to processes of care were identified following thematic analysis of the transcripts: relationships and communication; access and coordination; experience of discrete episodes of care; and psychological support. These impacted how care was experienced and, for some patients, outcomes of CHD and its treatment as well as broader health outcomes. In addition, context relating to stages of the patient journey were described, together with patient-related factors such as patients' knowledge and expertise in their own condition.

Conclusions

For peer review only - http://bmjopen.bmj.com/site/about/guidelines.xhtml

People with CHD and their parents/carers want individualised, person-centred care delivered within an appropriately resourced, multidisciplinary service. Although examples of excellent care were provided it is evident that, from the perspective of patients and parents/carers, some NHS Standards for people with CHD were not being met.

Strengths and limitations

- Data collection methods (asynchronous discussion forums on Facebook) facilitated accessibility for participants and resulted in a large sample of geographically and clinically diverse participants of different ages
- Use of social media to collect data supports participation by those who might otherwise not engage in research
- Most participants were female and ethnic diversity was limited
- The study design precluded participation from those who could not read English, use social media or were unfamiliar with, or without access to, appropriate technology

For peer review only

For peer review only - http://bmjopen.bmj.com/site/about/guidelines.xhtml

Background

Congenital heart disease is the most common congenital anomaly, with a global incidence of 6-8 per 1000 live births.[1] Successive improvements in surgical and medical care mean many more children with CHD now reach adulthood and live longer adult lives.[2 3] Furthermore, increasing numbers live with significant physical and mental health co-morbidities alongside the natural progression of their CHD.[4-7] The vast majority of this heterogeneous group in the UK and other developed countries are advised to remain under specialist life-long follow-up[8 9] though sporadic clinic non-attendance is frequent, particularly in adulthood[10], and patients may be lost to follow-up entirely, sometimes with significant consequences.[11] Unlike many other chronic conditions, organisation of care for these patients remains, for the vast majority, under the remit of the specialist team at the tertiary hospital, which may be far from where they live. In general, routine investigations are performed annually, sometimes more frequently in those with complex or advanced disease, and may include unanticipated invasive procedures.[10] Some may be seen in outreach clinics, usually by members of the specialist team but also by non-CHD cardiovascular specialists,[12] following a 'hub and spoke' model of delivering care, but these services remain under-developed.[13] Attendances in primary care and at A&E, in similar care structures, are higher than for the general population.[14] Occasionally, people with CHD remain undiagnosed until adulthood[15 16] when they typically present with symptoms.

The way in which patients (and their carers) experience care has a significant impact on their engagement with that care and the professionals delivering it.[17] In the National Health Service (NHS) Outcomes Framework[18] 'ensuring that people have a positive experience of care' was identified as a priority, recognising the consequences for safety and effectiveness. However, if the NHS is to provide optimal care that meets the needs and expectations of service users, it is important to understand what characterises 'good' care from the patient and their family's perspective.

For peer review only - http://bmjopen.bmj.com/site/about/guidelines.xhtml

In this study we elicited the perspectives of patients and carers about their experiences of CHD care and how that related to how patients and carers would like care to be delivered. This was carried out as part of a wider study (Congenital Heart Audit: Measuring Progress In Outcomes Nationally (CHAMPION))[19] aiming to develop tools for routine measurement of outcomes in CHD that are considered relevant to all stakeholders.

Methods

Design

A qualitative approach underpinned by an interpretivist framework was used, in which participant (patient or parent/carer) views were elicited through online discussion forums.

Patient and Public Involvement (PPI)

A co-researcher who was also a CHD patient (AC) led a PPI group for the overall study (comprising three adults with CHD and one grandparent of a child with CHD). The PPI group reviewed and provided feedback on content and language of questions for the online forum and on the findings prior to submission. Forum questions and presentation of the findings were revised based on feedback from the PPI group.

Participants and data collection

We adopted an established approach.[20 21 22] Three UK CHD charities (Little Hearts Matter; Children's Heart Federation; Somerville Foundation) facilitated and moderated one or more closed, asynchronous, online discussion groups via their Facebook pages. This enabled us to elicit a range of views (parents/carers of younger (<12 years) children, adolescents and adults; adolescents and adults with CHD) across the spectrum of CHD severity (complex (single ventricle conditions)/less complex (biventricular conditions)). The research team developed questions and revised content and language based on feedback from the PPI group and charity collaborators. On the advice of the

For peer review only - http://bmjopen.bmj.com/site/about/guidelines.xhtml

For peer review only

charities, separate forums were run for adults with CHD, adolescents with CHD and parents/carers of children and adolescents with CHD. Carers of adult patients participated in the adult discussion groups. Discussion forums were advertised by each charity on their web page with potential participants signposted to the charity's Facebook page for further information about the study. People wanting to participate were asked to provide basic demographic details and subsequently directed to the appropriate closed Facebook group where they could join the discussion. Enrolment was ongoing, such that new participants could join at any stage while the forum was running; they could see and respond to questions that had already been posted if they wished. Charities could introduce prompts or post new questions, based on responses. Questions for each forum were similar, with minor wording revisions to reflect the respondent group (Table 1). Forums ran for 12-24 weeks (during February-August 2021, dependent on responses and interruptions due to Covid-19). Charities were responsible for all day to day running and moderation of forums in line with a standard operating procedure (SOP) previously developed in collaboration with the research team.[21] The SOP included processes for managing inappropriate and/or offensive messaging and distressed users as well as procedures for the day to day running of the forum.

Data management and analysis

Identifiable data were removed by the charities before transfer of the anonymised transcript with aggregated demographics to the research team. Transcripts were thematically analysed[23] independently by three research team members (JW, FK, LC): codes were given to segments of data and similar codes grouped to create themes related to experience of CHD care. The researchers met to discuss and finalise themes. Themes and transcripts were sent to an additional research team member (not involved in coding) to review (SC) to ensure all data about experience of care were accurately represented in the themes.

For peer review only - http://bmjopen.bmj.com/site/about/guidelines.xhtml

Results

Five forums (two for adults with CHD, two for parents/carers and one for younger patients (<18 years of age) were run across three charities. In total 343 participants signed up to the forums Demographics of participants are shown in Table 2.

Four linked themes related to processes of care were identified: relationships and communication, access and coordination, experience of discrete episodes of care and psychological support. These impacted how care was experienced and, for some patients, outcomes of CHD and its treatment as well as broader health outcomes. Processes of care were described in different cardiac and community settings. In addition, context relating to stages of the patient journey, time of diagnosis and patient-related factors e.g. patients' expertise in their own condition were described (Figure 1). Themes are described below, with illustrative quotes from adults with CHD unless otherwise stated.

Relationships and communication

The concepts of relationships and communication were intertwined and evident throughout all forum groups. Participants discussed the importance of the patient-clinician relationship in terms of the characteristics of the clinician and of the relationship. People with CHD want to have a '*great relationship with [the] consultant, knowing you'll see someone who 'knows' you and your condition really well',* and emphasised the need for awareness of, and continuity in, those providing care: *"They don't have to be my mate but it's important that they're cognisant of the context because CHD is lifelong and affects all parts of my life."* The importance of professionals being kind, approachable, honest and caring as well as being excellent communicators and expert in CHD were highlighted. Participants felt they needed a team they respect and trust, who listen and treat them "*like a human being and not a burden on resources. The impact of being told that - both overtly and implicitly - is severe, and affects engagement."* A parent of a child said of the surgeon: *"His calm, kind, confident communication and how he took personal responsibility was pivotal in giving me the*

For peer review only

confidence to trust him to operate on my son." Another parent said*: "The best care we have received has been when we were listened to and not judged for the questions and requirements we had."* Adolescents with CHD commented on the value of clinicians being interested in them beyond their heart condition: "*When I was in children's I loved that my cardiologist took an interest in my life and asked about hobbies, school and social life. I feel if more people did this you would feel even more welcomed and calm and not nervous."* Other patients described the importance of being able to *"maintain a great relationship with the CNS* [clinical nurse specialist] *nurses as often it is hard to remember or say everything needed to the consultant,"* with the need for others (staff and/or family members) to advocate for them when they feel unable to do so also acknowledged. Recognition that adults with CHD are experts in their own care and need to feel empowered and believed was highlighted, with emphasis on the importance of feeling part of the team: *"I like the feeling that my medical care is a partnership between the cardiac team (who know about hearts) and me (I know about me and what's normal/ok/priority for me)."* Another commented: *"I hope the team understands a little of what I've been through, what I bring with me in terms of experience, knowledge but also emotionally."* Participants also discussed the importance of being involved in decision making, *"What's important to me is that all my team totally understand my condition, listen to me and include me in any decisions".* One participant talked about being able to make *"Self-referrals, avoiding local cardiac care, we know when we feel shitty".* Some participants also discussed occasions when they had been admitted to local hospitals where they were not known and communication was poor, with consequences for broader health outcomes: "*My local district general hospital also scared the pants off me after being admitted with palpitations when a cardiologist with 'an interest In GUCH' decided to hint on my life expectancy...something I'd never asked for and which has damaged my mental health ever since*".

Practical aspects of communication with professionals were discussed in terms of functional elements (e.g. timeliness), content (e.g. information about procedures and associated risks, accuracy

For peer review only

of content) and the mechanisms of communication: *"…results from tests being given by consultant not just a short letter that is sent to GP".* Decision making was also discussed in relation to the importance of being given honest feedback: *"I'd rather have the detail with an explanation about what this means even though it might be difficult to come to terms with. I want to be making informed decisions."*

Many people with CHD are under the care of multiple teams and specialists and participants described the importance (and often failure) of communication between professionals to ensure joined up care – *"Could that all have been avoided if the surgical team had involved cardiology more? Better communication certainly would have helped either way"* – and discussed communication between teams – "*why do the ACHD team and the EP team not seem to talk to one another? What's the point of coming up with a plan with the one team and then the other walking in and saying something different?"* They also described challenges in communication between specialist centres and outreach and primary care services. As one participant said, *"I'm seen at an outreach clinic and there is quite often a lack of communication between the outreach clinic and the main hospital"* whilst another described the importance of there being "*a note on the GP system so that when you appear with heart symptoms they take it seriously"*. In contrast, a mother of a paediatric patient commented*: "I feel very privileged to have such a joined-up way of all the hospitals and primary care teams working together for my son's health".*

Parents of paediatric patients discussed the importance of communication with schools and nurseries and getting help with education, health and care plans: *"Schools always panic over everything. My son's first week back at school I was called 3 times by the matron due to little things. I asked cardiac nurses for info for school and was just given general information leaflets. They should be more person-centred as 2 kids with the same condition will be at different stages and have different symptoms."*

For peer review only - http://bmjopen.bmj.com/site/about/guidelines.xhtml

Access and coordination

Respondents described challenges accessing care when they were moving to another hospital, out of hours or when they were unwell or needed advice: *"Knowing that I can ask a question or get a query answered quickly is very important to me. So, timely and accessible care is important*". Some people with CHD had been lost to follow-up, sometimes with a significant impact on their health outcomes: "*my checkups were not continued…notes must have been lost somewhere. I had no drugs, nothing and I had a stroke which I believe could have been avoided"* or had to fight to get treatment: *"Actually getting any care at all would be good… I haven't been referred to the ACHD team despite asking and my 'care' has been non-existent".* Others talked about "*being lost in the system…never followed-up*" or a lack of appropriate action when they were seen: "*Saw him [consultant] once then nothing happened. No blood thinner etc. I had a stroke and am so bitter…my life is in tatters due to lack of minimal care*". In contrast, some parents talked about the ready access they had to the clinical team or an open-door policy to access the ward if their child was unwell: *"My son is still looked after [by] a multidisciplinary team over two hospitals and we have 24 hour access to a local hospital that also have wonderful and caring doctors. My GP called us at the various hospitals to check we were ok."*

Coordination of care was mentioned frequently, particularly in relation to appointments and investigations and organising them to happen at the same time ("*Appointments in a cluster – MRI, echo etc so we don't have multiple trips to manage"),* with some highlighting examples of good practice: *"In recent years (pre-Covid) it has gone like clockwork and I'm usually in hospital for a couple of hours at most."* Others felt that changes to clinic arrangements made during Covid-19 were positive and should be continued: "*What has been useful in Covid times is going for tests and then having a video or telephone conversation to discuss those test results. I think this is a good model to take forward*." Others talked about the complexity of their problems and needing

For peer review only - http://bmjopen.bmj.com/site/about/guidelines.xhtml

everything to be together: “*I would like somewhere to go where all our older problems are taken care of…heart, everything under one roof, not non-guch [ACHD]* hosp where docs don't understand your condition”.* Another respondent said: “*Definitely not having consultant appointment- test arranged for 2 months later, another test a couple of months after that and because of that result another test after that with a consultation several months after that which takes you a year down the line to then have a consultation with someone who hasn't even looked at the results of the tests!”*

(*Patients often refer to GUCH, which stands for grown-up congenital heart disease, now referred to as ACHD or adult congenital heart disease).

Experience of discrete episodes of care

Participants discussed experiences of clinics and inpatient stays, describing the importance of being given enough time (*“appointments not to be rushed”),* being made to feel “*at home…and calm*” (adolescent), their experiences of undergoing tests and the people conducting them, and what makes attending difficult. Experience of inpatient stays was described in terms of the environment, people working on the ward (particularly in relation to staff changeover at the end of a shift and whether staff had read their medical notes: “*We find every time my son is admitted to hospital the staff do not seem to read his notes and start to panic that he has an abnormal heart rhythm. Also seeing different doctors each day and them saying different things*”), ability to sleep on the ward, available activities (children) and experience of discharge: *“Having a good discharge! Why oh why can it take hours and hours to get out of hospital when you are clinically well enough to leave. Getting out of hospital is as important as being admitted in the first place.”* Respondents highlighted the value of being routinely asked about their experiences of outpatient and inpatient care through completion of patient-reported experience measures and the importance of telling patients what has been or will be done with the information. Linked to this, some adults with CHD articulated the importance of patients being involved in service development initiatives.

For peer review only - http://bmjopen.bmj.com/site/about/guidelines.xhtml

Psychological support

Mental health and the need for psychological support were mentioned as important but often lacking. Some described waiting for psychology support for many years or services not being available at all: *"Access to psychology at clinics – that is non-existent"*, a scenario also reported in paediatric services: *"Lack of ongoing psychology support – we have waited 4 years"* (parent). Some adults received a late diagnosis, with repercussions for their mental health: *"I found out later in life and I found the mental health support was limited, specifically with adjusting to the news that my heart could have ripped itself apart. Those who are diagnosed later or suddenly escalate should be referred to mental health specialists as a priority."* Another participant who had experienced psychological issues for which they had not received any support commented: *"My story exposed the importance of psychological care and the potential physical damage that can be caused to the cardiological system from stress, anxiety and childhood trauma instigated by CHD."* The importance of other professionals in the wider team was also identified, including for children – *"I think that every child that had life-long illness should have regular play therapy, to help deal with their fear and emotions they face."* Finally, the valuable role of patient support organisations was acknowledged, *"They put me in touch with [charity], who have been my saviour."*

Discussion

In this study our aim was to understand the perspectives of people with CHD and their parents/carers on their experiences of care and how that related to the ways in which they would like care to be delivered and, as part of a wider project, outcomes evaluated. Four key themes were identified (relationships and communication, access and coordination, experiences of discrete episodes of care and psychological support) with participants providing examples of when things went well and less well. The findings support those of previous research with people with CHD[24-29] as well as other groups of people with chronic illness,[30-32] in which the importance of care being

For peer review only - http://bmjopen.bmj.com/site/about/guidelines.xhtml

delivered by competent clinicians, effective patient-clinician communication, coordination of and access to care and the need for psychological support have all been highlighted. However, of concern, is that the findings suggest that a number of the NHS CHD recommended standards and service specifications[33] are presently not being met, e.g. psychology provision and referral to ACHD services – although there are examples of excellent care, particularly in paediatric services. Furthermore, the findings indicate that, contrary to the NHS vision of promoting equality and reducing health inequities, service delivery is variable between centres in the UK.

There was a degree of overlap between themes, particularly between the themes of 'relationships and communication' and 'access and coordination'. The importance of relationships between patients and clinicians is well documented.[29 34 35] People with CHD often have complex diagnoses and patients highlighted how they wanted professionals to really know about their specific diagnosis and treatment pathway. Communication is part of this, not only between patients and professionals but also between different professionals as these patients are often under multiple specialities or seen in several centres. This also links to coordination of care and the need for a more joined up approach. The majority of our participants were adult patients and much of what they reported supports the view that the current way in which ACHD ambulatory care is delivered in the UK (the hub and spoke approach)[13] does not provide either equitable or patient-centred care.[36] Poor communication and coordination of care also resulted in some people with CHD being lost to follow-up, particularly at times of transition from paediatric to adult services or from one hospital to another, or suffering potentially avoidable complications, with consequences for longer term health outcomes. Whilst these findings are not new, the complexity of this patient cohort and the requirement for life-long follow-up increase their vulnerability to poorer outcomes and experiences.

Building on the conceptual map of Entwistle[37] who undertook a critical interpretive synthesis of research literature on patients' perspectives of health care delivery, we have used our findings to

For peer review only - http://bmjopen.bmj.com/site/about/guidelines.xhtml

characterise what 'good care' looks like for people with CHD and their carers (Figure 2). This approach recognises that the characteristics of health services and actions of professionals impact how patients (and families) experience and engage with care and our findings resonate with previous studies.[38] People with CHD are a unique group due to their complexity, the requirement for life-long care and the diversity in how and by whom care is delivered. The NHS Standards for patients with CHD[33] clearly outline what centres should provide in the way of services and access to professionals and this study builds on these by offering insight from the service users' perspective.

Limitations

Data collection (asynchronous discussion forums on Facebook) facilitated accessibility for participants and resulted in geographically and clinically diverse participants of different ages. However, a broad range of ethnicities was not seen and the vast majority of participants were female, consistent with previous findings that females are more likely to use social media.[39 40] The study design precluded participation from those who could not read English, use social media or were unfamiliar with, or without access to, appropriate technology and potentially those with intellectual, physical and/or sensory disability. Patients and families already in contact with the charities may have been more likely to engage, leading to potential selection bias and limited generalisability of the findings. Due to the format of the data provided we were unable to link demographic characteristics to responses and it was not always possible to determine how many participants contributed to discussions rather than just signing up to the forum. Inevitably views of more vocal participants may be over-represented although careful reading of the transcript did enable us to determine to some extent posts from the same participant. Finally, some adults with CHD experienced elements of the patient journey some time ago and their experiences may not be reflective of how some of these aspects of care are managed today.

For peer review only

For peer review only - http://bmjopen.bmj.com/site/about/guidelines.xhtml

The fourth principle of the NHS Constitution of England[41] states that 'the patient will be at the heart of everything the NHS does' and that 'NHS services must reflect, and should be coordinated around and tailored to, the needs and preferences of patients, their families and their carers.' Delivering good care and a good patient experience are crucial if patients are to engage, and remain engaged, with their health care and outcomes are to be optimised;[42] understanding what 'good' care means to patients and families is a key element of that process. Our findings, whilst indicating that people with CHD share commonalities with other patient groups, also reflect the challenges of delivering comprehensive care to this diverse and growing population. Participants have articulated how they would like care to be delivered and what good care looks like, with an emphasis on individualised, person-centred care delivered within an appropriately resourced, multidisciplinary service. They want to be acknowledged as experts in their own condition and for the whole life impact of CHD and its treatment to be recognised, not just the physical impact.

Future directions and conclusion

People with CHD and their parents/carers know how they want care to be delivered and yet, as is evident from the resonance of our findings with those of others, the gap between what patients want (and have a right to expect) and what they receive remains wide. The introduction of standards for the delivery of care to patients with CHD and their families has clearly not resulted in all of those standards being met or in the delivery of equitable care. Patient views need to inform quality improvement initiatives or, recognising that what patients want may not always be deliverable and may remain aspirational, patients' and families' expectations need to be managed. Patients want their voices to be heard and using the results to inform the development of patient-reported experience measures for the CHD population, for routine completion, would enable services to be continually evaluated, benchmarked and improved, with resulting benefits for optimising patients' experiences and outcomes and the opportunity to reduce inequity in health service delivery.

For peer review only - http://bmjopen.bmj.com/site/about/guidelines.xhtml

Acknowledgements

We wish to acknowledge the invaluable contribution to the CHAMPION project of Dr Adrian Chester, a highly respected researcher, committed patient advocate, colleague and friend who sadly died in November 2023.

The authors thank The Somerville Foundation, Little Hearts Matter and the Children's Heart Federation for recruiting participants and moderating the forums, and for their contributions, together with the PPI group, to the development and review of the forum questions.

Research at Great Ormond Street Hospital is supported by the Great Ormond Street Hospital NIHR Biomedical Research Centre.

Funding statement

This study is independent research funded by the National Institute for Health and Care Research (Policy Research Programme, Congenital Heart Audit: Measuring Progress In Outcomes Nationally (CHAMPION), PR-R20-0318-23001). The views expressed in this publication are those of the author(s) and not necessarily those of the NHS, the National Institute for Health Research or the Department of Health and Social Care.

Competing interest statement

None of the authors have any conflicts of interest to declare.

Contributorship statement

JW – contributed to the design of the study and undertook the initial analysis of the transcripts; wrote the initial draft of the manuscript and approved the final version.

For peer review only

CP – contributed to the design of the study; revised the manuscript and approved the final version.

LC – contributed to the design of the study and undertook the initial analysis of the transcripts; revised the manuscript and approved the final version.

AC – contributed to the design of the study and led the study PPI; revised the manuscript and approved the initial submitted version.

FK – contributed to the design of the study and undertook the initial analysis of the transcripts; revised the manuscript and approved the final version.

SC – contributed to the design of the study; independently checked that all data related to experience of care were represented appropriately in the themes; revised the manuscript and approved the final version.

Data sharing statement

All data relevant to the study are included in the article.

Ethics approval statement

The study was granted ethical approval by the North of Scotland Research Ethics Service (ref: 20/NS/0022).

For peer review only

References

1. van der Linde D, Konings EE, Slager MA, et al. Birth prevalence of congenital heart disease worldwide: a systematic review and meta-analysis. *J Am Coll Cardiol* 2011;58(21):2241-7. doi: 10.1016/j.jacc.2011.08.025 [published Online First: 2011/11/15]
2. Global Burden of Disease Congenital Heart Disease Collaborators. Global, regional, and national burden of congenital heart disease, 1990-2017: a systematic analysis for the Global Burden of Disease Study 2017. *Lancet Child Adolesc Health* 2020;4(3):185-200. doi: 10.1016/S2352-4642(19)30402-X [published Online First: 2020/01/25]
3. Benziger CP, Stout K, Zaragoza-Macias E, et al. Projected growth of the adult congenital heart disease population in the United States to 2050: an integrative systems modeling approach. *Popul Health Metr* 2015;13:29. doi: 10.1186/s12963-015-0063-z [published Online First: 2015/10/17]
4. Desai R, Patel K, Dave H, et al. Nationwide Frequency, Sequential Trends, and Impact of Co-morbid Mental Health Disorders on Hospitalizations, Outcomes, and Healthcare Resource Utilization in Adult Congenital Heart Disease. *Am J Cardiol* 2020;125(8):1256-62. doi: 10.1016/j.amjcard.2020.01.024 [published Online First: 2020/02/23]
5. El-Chouli M, Meddis A, Christensen DM, et al. Lifetime risk of comorbidity in patients with simple congenital heart disease: a Danish nationwide study. *Eur Heart J* 2022 doi: 10.1093/eurheartj/ehac727 [published Online First: 2022/12/09]
6. Brida M, Lovric D, Griselli M, et al. Heart failure in adults with congenital heart disease. *Int J Cardiol* 2022;357:39-45. doi: 10.1016/j.ijcard.2022.03.018 [published Online First: 2022/03/15]
7. Alonso-Gonzalez R, Borgia F, Diller GP, et al. Abnormal lung function in adults with congenital heart disease: prevalence, relation to cardiac anatomy, and association with survival. *Circulation* 2013;127(8):882-90. doi: 10.1161/CIRCULATIONAHA.112.126755 [published Online First: 2013/02/06]
8. Kempny A, Diller GP, Dimopoulos K, et al. Determinants of outpatient clinic attendance amongst adults with congenital heart disease and outcome. *Int J Cardiol* 2016;203:245-50. doi: 10.1016/j.ijcard.2015.10.081 [published Online First: 2015/11/01]
9. Baumgartner H, Budts W, Chessa M, et al. Recommendations for organization of care for adults with congenital heart disease and for training in the subspecialty of 'Grown-up Congenital Heart Disease' in Europe: a position paper of the Working Group on Grown-up Congenital Heart Disease of the European Society of Cardiology. *Eur Heart J* 2014;35(11):686-90. doi: 10.1093/eurheartj/eht572 [published Online First: 2014/01/30]
10. Coats L, Chaudhry B. Ambulatory Care in Adult Congenital Heart Disease-Time for Change? *J Clin Med* 2022;11(7) doi: 10.3390/jcm11072058 [published Online First: 2022/04/13]
11. Wray J, Frigiola A, Bull C, et al. Loss to specialist follow-up in congenital heart disease; out of sight, out of mind. *Heart* 2013;99(7):485-90. doi: 10.1136/heartjnl-2012-302831 [published Online First: 2012/12/22]
12. Seidel L, Nebel K, Achenbach S, et al. Facts about the General Medical Care of Adults with Congenital Heart Defects: Experience of a Tertiary Care Center. *J Clin Med* 2020;9(6) doi: 10.3390/jcm9061943 [published Online First: 2020/06/26]
13. Department of Health. The UK strategy for rare diseases, 2013.
14. Verheugt CL, Uiterwaal CS, van der Velde ET, et al. The emerging burden of hospital admissions of adults with congenital heart disease. *Heart* 2010;96(11):872-8. doi: 10.1136/hrt.2009.185595 [published Online First: 2010/04/22]
15. De Angelis F, Savino K, Colombo A, et al. Never too Grown-Up for a Congenital Heart Disease: Diagnosis of Transitional Atrioventricular Canal in a 50-Year-Old Male. *J Cardiovasc Echogr* 2019;29(1):35-38. doi: 10.4103/jcecho.jcecho_28_18 [published Online First: 2019/04/23]

For peer review only - http://bmjopen.bmj.com/site/about/guidelines.xhtml

16. Sadeghpour A, Kim H, Chamis AL. Undiagnosed Atrial Septal Defect in the Setting of Comorbidities and Ventricular Failure: Seemingly Simple Disease with a Challenging Diagnosis. *CASE (Phila)* 2023;7(2):72-80. doi: 10.1016/j.case.2022.09.010 [published Online First: 2023/03/03]
17. de Silva D. Measuring patient experience: The Health Foundation 2013.
18. Department of Health. NHS Outcomes Framework 2012/13., 2011.
19. Crowe S, Chester A, Ambler G, et al. Congenital Heart Audit: Measuring Progress In Outcomes Nationally (CHAMPION) 2019 [Available from: https://fundingawards.nihr.ac.uk/award/PR-R20-0318-23001.
20. Rajagopal V, Brown K, Pagel C, et al. Parental understanding of our communication of morbidity associated with paediatric cardiac surgery: a qualitative study. *BMJ Paediatr Open* 2020;4(1):e000578. doi: 10.1136/bmjpo-2019-000578 [published Online First: 2020/03/11]
21. Wray J, Brown K, Tregay J, et al. Parents' Experiences of Caring for Their Child at the Time of Discharge After Cardiac Surgery and During the Postdischarge Period: Qualitative Study Using an Online Forum. *J Med Internet Res* 2018;20(5):e155. doi: 10.2196/jmir.9104 [published Online First: 2018/05/11]
22. Wray J, Pagel C, Chester AH, et al. What was the impact of the first wave of COVID-19 on the delivery of care to children and adults with congenital heart disease? A qualitative study using online forums. *BMJ Open* 2021;11(9):e049006. doi: 10.1136/bmjopen-2021-049006 [published Online First: 2021/10/02]
23. Braun V, Clarke V. Using thematic analysis in psychology. *Qualitative research in psychology* 2006;3:77-101.
24. Wijayarathne PM, Ng A, Menahem S. How do Adults With Congenital Heart Disease Evaluate Their Clinical Care? *World J Pediatr Congenit Heart Surg* 2023;14(1):55-62. doi: 10.1177/21501351221127895 [published Online First: 2023/02/28]
25. van Staa A, Jedeloo S, van der Stege H, et al. "What we want": chronically ill adolescents' preferences and priorities for improving health care. *Patient Prefer Adherence* 2011;5:291-305. doi: 10.2147/PPA.S17184 [published Online First: 2011/07/28]
26. Mackie AS, Bravo-Jaimes K, Keir M, et al. Access to Specialized Care Across the Lifespan in Tetralogy of Fallot. *CJC Pediatr Congenit Heart Dis* 2023;2(6Part A):267-82. doi: 10.1016/j.cjcpc.2023.09.004 [published Online First: 2024/01/02]
27. Lopez KN, Baker-Smith C, Flores G, et al. Addressing Social Determinants of Health and Mitigating Health Disparities Across the Lifespan in Congenital Heart Disease: A Scientific Statement From the American Heart Association. *J Am Heart Assoc* 2022;11(8):e025358. doi: 10.1161/JAHA.122.025358 [published Online First: 2022/04/08]
28. Kovacs AH, Brouillette J, Ibeziako P, et al. Psychological Outcomes and Interventions for Individuals With Congenital Heart Disease: A Scientific Statement From the American Heart Association. *Circ Cardiovasc Qual Outcomes* 2022;15(8):e000110. doi: 10.1161/HCQ.0000000000000110 [published Online First: 2022/07/22]
29. Lammers AE, Helm PC, Bauer UM, et al. Optimizing Care for Adults with Congenital Heart Disease: Results of a Conjoint Analysis Based on a Nationwide Sample of Patients Included in the German National Register. *J Clin Med* 2021;10(16) doi: 10.3390/jcm10163483 [published Online First: 2021/08/28]
30. Anderson R, Barbara A, Feldman S. What patients want: A content analysis of key qualities that influence patient satisfaction. *J Med Pract Manage* 2007;22(5):255-61. [published Online First: 2007/05/15]
31. Simpson A, Bloom L, Fulop NJ, et al. How are patients with rare diseases and their carers in the UK impacted by the way care is coordinated? An exploratory qualitative interview study. *Orphanet J Rare Dis* 2021;16(1):76. doi: 10.1186/s13023-020-01664-6 [published Online First: 2021/02/12]

For peer review only - http://bmjopen.bmj.com/site/about/guidelines.xhtml

32. Hannane A, Misane L, Devouassoux G, et al. Asthma patients' perception on their care pathway: a qualitative study. *NPJ Prim Care Respir Med* 2019;29(1):9. doi: 10.1038/s41533-019-0121-2 [published Online First: 2019/04/04]
33. NHS England. Congenital Heart Disease Standards & Specifications 2016 [Available from: https://www.england.nhs.uk/wp-content/uploads/2018/08/Congenital-heart-disease-standards-and-specifications.pdf.
34. Kelley JM, Kraft-Todd G, Schapira L, et al. The influence of the patient-clinician relationship on healthcare outcomes: a systematic review and meta-analysis of randomized controlled trials. *PLoS One* 2014;9(4):e94207. doi: 10.1371/journal.pone.0094207 [published Online First: 2014/04/11]
35. Ridd M, Shaw A, Lewis G, et al. The patient-doctor relationship: a synthesis of the qualitative literature on patients' perspectives. *Br J Gen Pract* 2009;59(561):e116-33. doi: 10.3399/bjgp09X420248 [published Online First: 2009/04/04]
36. Chaudhry I, Ghassani A, Wray J, et al. Outpatient care of adults with congenital heart disease in the UK: a qualitative appraisal of the clinician perspective. *Open Heart* 2024;11(1) doi: 10.1136/openhrt-2023-002420 [published Online First: 2024/01/30]
37. Entwistle V, Firnigl D, Ryan M, et al. Which experiences of health care delivery matter to service users and why? A critical interpretive synthesis and conceptual map. *J Health Serv Res Policy* 2012;17(2):70-8. doi: 10.1258/jhsrp.2011.011029 [published Online First: 2011/10/05]
38. Robert G, Cornwell J. Rethinking policy approaches to measuring and improving patient experience. *Journal of Health Services Research & Policy* 2013;18(2):67-69. doi: 10.1177/1355819612473583
39. Ye L, Chen Y, Cai Y, et al. Gender Differences in the Nonspecific and Health-Specific Use of Social Media Before and During the COVID-19 Pandemic: Trend Analysis Using HINTS 2017-2020 Data. *J Health Commun* 2023:1-10. doi: 10.1080/10810730.2023.2193151 [published Online First: 2023/03/22]
40. Johnson LA, Dias N, Clarkson G, et al. Social media as a recruitment method to reach a diverse sample of bereaved parents. *Appl Nurs Res* 2019;50:151201. doi: 10.1016/j.apnr.2019.151201 [published Online First: 2019/11/05]
41. Department of Health and Social Care. Guidance - The NHS Constitution for England 2021 [Available from: https://www.gov.uk/government/publications/the-nhs-constitution-for-england/the-nhs-constitution-for-england.
42. Doyle C, Lennox L, Bell D. A systematic review of evidence on the links between patient experience and clinical safety and effectiveness. *BMJ Open* 2013;3(1) doi: 10.1136/bmjopen-2012-001570 [published Online First: 2013/01/08]

For peer review only

Table 1: Examples of questions related to experiences of care for the adult patient forums

Questions for the parent/carer and teenager forums were similar, with minor wording changes to reflect those respondent groups (e.g. designed to appeal to teenagers or wording appropriate for carers rather than patients). Charities could introduce questions to probe further – e.g. can you tell us more about a clinic appointment that went well or less well?

1. Understanding how patients and families judge how 'good' a service is will help us to identify things that services do well and those aspects where the NHS could improve.

    - What do you think makes a service good quality? What sort of things do you want to know about a hospital or service to judge whether it is a good place to care for you?
    - What things might make you worry that the hospital / service was not as good quality as other hospitals / services?

2. We would like to know about what information you think should be collected about people's *experience* of the care they receive – for example, about how health professionals talk to patients and their families.

    - Thinking about some recent care you have had, are there any particular aspects of your experience that the NHS could collect about those services to judge their quality?

3. We would like to ask you about your experience of attending clinic appointments, which we recognise is not always easy for patients. How easy or difficult have you found it to keep up with follow-up appointments? Are there particular things that make it easy or difficult for you to attend follow-up appointments? How easy do you find it to see professionals other than doctors and nurses when you attend clinic, if you want to (such as social workers, psychologists, play specialists)?

4. We'd like to think now about some particular things that may happen to a patient with CHD. We know that patients with CHD often have to undergo a number of medical interventions, such as catheters and heart surgery. We are interested in understanding how patients and families judge whether an operation or catheter has gone well as this will help us to identify areas where hospitals can improve the service they provide.

    - Have you had a medical intervention for CHD (surgery or a catheter procedure) since you have been in adult services? If so, thinking back to that time, can you remember what was most important to you about the intervention and the care you received?
    - Making the decision to agree to an operation or catheter can be a big step. Do you think you were given enough information to make the decision? Do you remember what you were told about the likely outcomes of the procedure and how it might affect you?
    - And what about now? Thinking about that medical intervention, are there other things that are important to you now about how it went and any impact it has had? Were your expectations about your surgery met, both at the time and now?

Table 2: Characteristics of those who signed up to the online forums (n=343)

	Number (%)
Participants: Adults with CHD	235 (69)
Young people with CHD	11 (3)
Parents/carers of adult patients with CHD	7 (2)
Parents/carers of children with CHD	90 (26)
Participant gender: Male	33 (10)
Female	245 (71)
Unknown	65 (19)
Participant age group: <16 years	2 (1)
16-20	11 (3)
21-30	32 (9)
31-40	69 (20)
41-50	72 (21)
51-60	65 (19)
61+ years	15 (4)
Unknown	77 (22)
Age group of person with CHD: 0-1 years	9 (3)
2-5 years	17 (4)
6-10 years	15 (4)
11-15 years	8 (2)
16-18 years	9 (3)
19+ years	238 (69)
Unknown	47 (14)
Participant ethnicity: White	326 (95)
Non-white	5 (1)
Unknown	12 (3)
Location of specialist service: England (North East)	11 (3)
England (North West)	26 (6)
England (Yorkshire and the Humber)	20 (6)
England (East Midlands)	14 (4)
England (West Midlands)	47 (14)
England (East of England)	9 (3)
England (London)	75 (22)
England (South East)	28 (8)
England (South West)	27 (8)
Wales	8 (2)
Scotland	24 (7)
Northern Ireland/other	15 (4)
Unknown/none	39 (11)
Location of home: England (North East)	13 (4)
England (North West)	43 (13)
England (Yorkshire and the Humber)	22 (6)
England (East Midlands)	20 (6)
England (West Midlands)	36 (10)
England (East of England)	23 (7)
England (London)	22 (6)
England (South East)	57 (17)
England (South West)	46 (13)

For peer review only - http://bmjopen.bmj.com/site/about/guidelines.xhtml

Wales	21 (6)
Scotland	25 (7)
Northern Ireland/other	8 (2)
Unknown	7 (2)
Complexity of CHD: Single ventricle condition	121 (35)
Biventricular condition	216 (63)
Unknown	6 (2)

A number of participants chose not to provide some or any demographic information

For peer review only

Figure legends

Figure 1: Map of the themes developed from the data

Figure 2: 'Ideal' characteristics and actions of health care services and staff and resulting potential for delivery of 'good' care

For peer review only

For peer review only - http://bmjopen.bmj.com/site/about/guidelines.xhtml

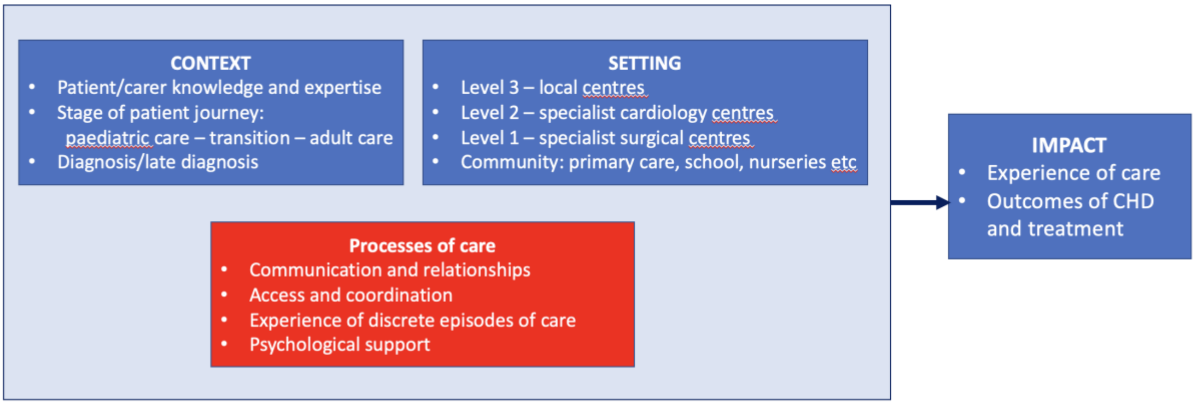

For peer review only

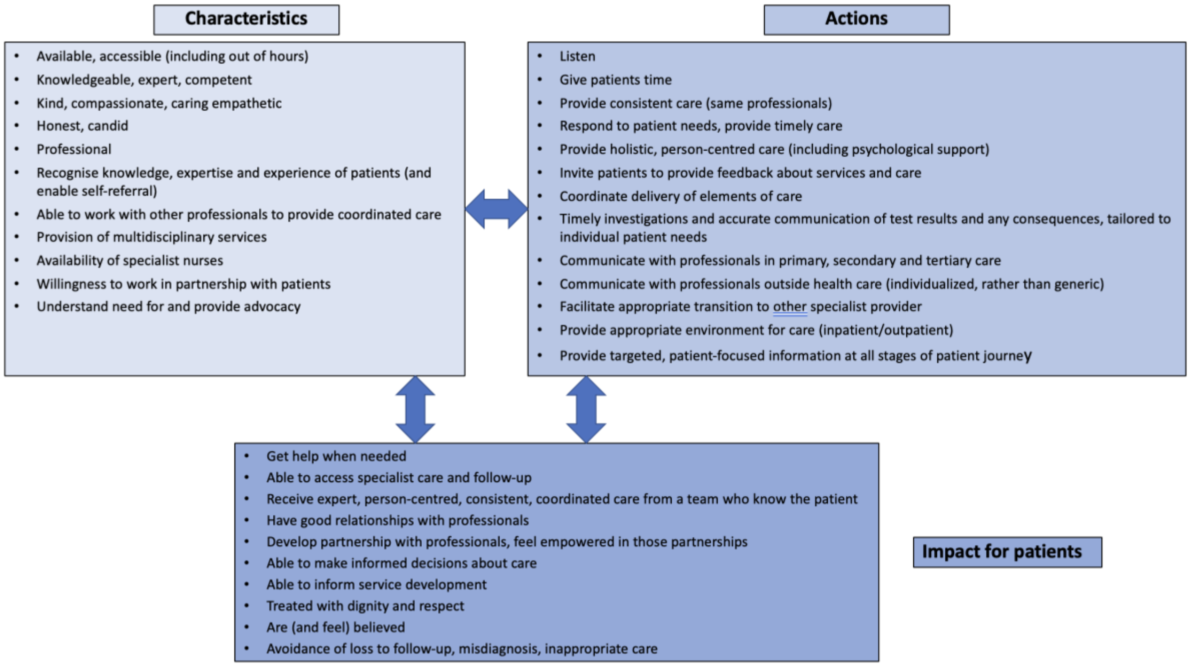

For peer review only
